# Pedestrianism

**Published:** 1879-04

**Authors:** 


### PEDESTRIANISM.

The American is capable of being influenced in the queerest ways. He is the most gregarious of animals. Get a crowd of him started after any absurdity, and his number can only be estimated by the capacity of the building to hold those that are sure to follow. Like the character in Mark Twain's jumping frog, he is always ready to bet on anything. Should a skipping contest between a lot of lively fleas be advertised, he would be there to wager his fortune upon the ability of a native insect over all competitors of a foreign variety. This proclivity cropped out in a most wonderful manner at the recent walking match at Gilmore's Garden. It really seems marvellous that $54,314.40 could be realized out of such an entertainment, and the excitement incident thereto kept up during an entire week. Stranger still that this furore should not be confined to New York, but that it became the absorbing theme of conversation for the people, giving inspiration for editorials, lectures, sermons, and telegraphic dispatches throughout the entire country. The school children discussed it, betting their marbles on their favorites—merchants, doctors, lawyers, everybody sought their daily papers, reading greedily the news from the match. To all this we make no special objection; but of what benefit (save to those engaged in the contest), was this trial of endurance? If a desire for physical culture had been stimulated throughout the country, some reason for rejoicing would remain. We heartily approve of athletic sports tending in that direction. But a six day's walk is not designed for, nor can it be designated "physical culture." It is simply a matter of endurance, an effort to ascertain who can first exhaust the store of reserve force and power laid up in every man's body against the day of sickness, or a call for unusual physical tax. Such feats are neither beneficial nor creditable. Months, even years will be required to restore the equilibrium in those who participated in that senseless walk. In the meantime, should sickness overtake any of them, they will surely die; because there is no reserve power left to aid them in such an emergency.

A still worse call upon this stored energy, occurs in walking fifteen hundred quarter-miles in the same number of quarter-hours. As we have laws for prevention of cruelty to animals, it certainly should be enforced in the case of these creatures, who are incapable of caring for their own bodies.

No healthier exercise can be indulged in than pedestrianism, but when it is abused to the extent of using it as a means of measuring man's endurance, then it becomes the most harmful of occupations, certain to destroy the strength and health of all who participate in it.

